# Supplementary material for: Capsaicin Inhibits Inflammation and Gastric Damage during H pylori Infection by Targeting NF-kB–miRNA Axis
Source: Pathogens. 2022 Jun 1;11(6):641. doi: 10.3390/pathogens11060641 (PMC9227394; doi:10.3390/pathogens11060641)
Supplement: Supplementary file 1 [file pathogens-11-00641-s001.zip › pathogens-1694696-supplementary.pdf]

Supplementary

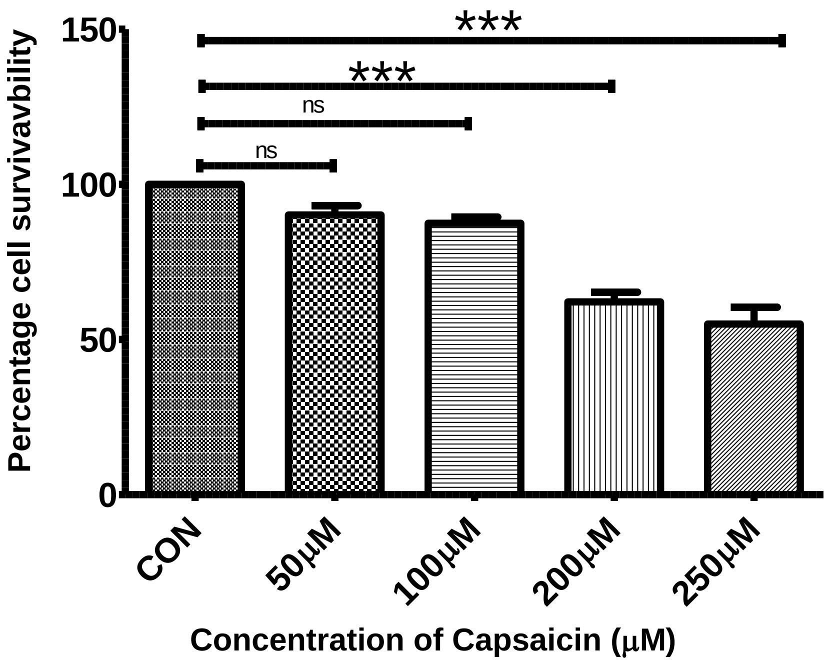

Figure S1: Viability assay of Capsaicin. Capsaicin was applied to AGS cells at different concentrations (50µM, 100µM, 200µM, 250µM) and percentage cell survivability was calculated. Data is represented as mean ± SEM. Values as tested by ANOVA followed by Tukey's post hoc test. '*'; '**'; '***'; represents significant difference between groups at $p < 0.05$, $p < 0.01$, $p < 0.001$ respectively. Experiments were performed in triplicates. Non-significant difference is represented as ns.

Table S1: List of Anti-bodies utilized.

Antibody	Company and Catalogue number
NF-κB p65	Santa Cruz (F-6), sc8008, Lot# C2619
Akt 1/2/3	Abcam, ab12681, Lot: GR8432
P-Akt	Abcam, ab81283, Lot: GR2400
P-NF-κB	Cell Signaling, CS#3033, Ser536, 93HI
α-Tubulin	BioBharti, #BB-ab0113
E-Cadherin	GeneTex, GTX629
CagA	Santa Cruz, sc-28368, Lot# F1421
β-actin	Santa Cruz, sc-47778, Lot# C1921

Table S2: List of Primers utilized.

Gene	Forward Primer	Reverse Primer
GAPDH (Mouse)	5′-GATCTTCGACAAGGGAGCTAAA-3′	5′-TCGCATTCTTCTACACGATAACA-3′
IL-6 (Mouse)	5′-GATAAGCTGGAGTCACAGAAGG-3′	5′-TTGCCGAGTAGATCTCAAAGTG-3′
IL-1β (Mouse)	5′-GGTGTGTGACGTTCCCATTA-3′	5′-TCGCATTCTTCTACACGATAACA-3′

TNFα (Mouse)	5′-CTGAGTTCTGCAAAGGGAGAG-3′	5′- CCTCAGGGAAGAATCTGGAAAG -3′
IL-10 (Mouse)	5′-GGGGTCATGGTGAGCACTAC-3′	5′-TGGGGGAAGTGGGTAAGAGT -3′
NLRP3 (Mouse)	5′-TCGGCCTTCTTCTTGTTCTG -3’	5′-GTTGTTCCACATCTCTCCTAGTT-3′
MsmiR21	Stem loop –<br>5′-GCCTAGCTTATCAGACTGA-3′<br><br>5′-GTCGTATCCAGTGCAGGGTCCGAGG TATTCGCACTGGATACGACTCAACAT-3′	Stem loop –<br>5′-GTGTCGTGGAGTCGGCAA-3′<br><br>Universal Reverse Primer<br>5′-GCCTAGCTTATCAGACTGA-3′
MsmiR223	Stem loop –<br>5′-TGGCTGTCAGTTTGTCAAAT-3′<br><br>5′-GTCGTATCCAGTGCAGGGTCCGAGG TATTCGCACTGGATACGACTGGGGT-3′	Stem loop –<br>5′-GTGTCGTGGAGTCGGCAA-3′<br><br>Universal Reverse Primer<br>5′-GCCTAGCTTATCAGACTGA-3′
MsmiRU6	Stem loop<br>5′-GCCAGCACATATACTAAAAT-3′<br><br>5′-CGCTTCACGAATTTGCGTGTCAT-3′	Stem loop<br>5′-CGCTTCACGAATTTGCGTGTCAT-3′<br><br>Universal Reverse Primer<br>5′-GCCTAGCTTATCAGACTGA-3′
H pylori 16S rDNA	5′-AGAGAAGCAATACTGTGAA-3′	5′-CGATTACTAGCGATTCCA-3′
